# Protein cleavage influences surface protein presentation in *Mycoplasma pneumoniae*

**DOI:** 10.1038/s41598-021-86217-y

**Published:** 2021-03-24

**Authors:** Iain J. Berry, Michael Widjaja, Veronica M. Jarocki, Joel R. Steele, Matthew P. Padula, Steven P. Djordjevic

**Affiliations:** 1 Ithree Institute, University of Technology Sydney, Broadway, PO Box 123, Ultimo, NSW 2007 Australia; 2 Proteomics Core Facility, University of Technology Sydney, Broadway, PO Box 123, Ultimo, NSW 2007 Australia; 3 School of Life Sciences, University of Technology Sydney, Broadway, PO Box 123, Ultimo, NSW 2007 Australia

**Keywords:** Proteolysis, Proteomics, Pathogens, Post-translational modifications

## Abstract

*Mycoplasma pneumoniae* is a significant cause of pneumonia and post infection sequelae affecting organ sites distant to the respiratory tract are common. It is also a model organism where extensive ‘omics’ studies have been conducted to gain insight into how minimal genome self-replicating organisms function. An N-terminome study undertaken here identified 4898 unique N-terminal peptides that mapped to 391 (56%) predicted *M. pneumoniae* proteins. True N-terminal sequences beginning with the initiating methionine (iMet) residue from the predicted Open Reading Frame (ORF) were identified for 163 proteins. Notably, almost half (317; 46%) of the ORFS derived from *M. pneumoniae* strain M129 are post-translationally modified, presumably by proteolytic processing, because dimethyl labelled neo-N-termini were characterised that mapped beyond the predicted N-terminus. An analysis of the N-terminome describes endoproteolytic processing events predominately targeting tryptic-like sites, though cleavages at negatively charged residues in P1′ (D and E) with lysine or serine/alanine in P2′ and P3′ positions also occurred frequently. Surfaceome studies identified 160 proteins (23% of the proteome) to be exposed on the extracellular surface of *M. pneumoniae*. The two orthogonal methodologies used to characterise the surfaceome each identified the same 116 proteins, a 72% (116/160) overlap. Apart from lipoproteins, transporters, and adhesins, 93/160 (58%) of the surface proteins lack signal peptides and have well characterised, canonical functions in the cell. Of the 160 surface proteins identified, 134 were also targets of endo-proteolytic processing. These processing events are likely to have profound implications for how the host immune system recognises and responds to *M. pneumoniae*.

## Introduction

*Mycoplasma pneumoniae* is an obligate parasite of the lower respiratory tract and the causative agent of atypical pneumonia in humans^1^. However, it is also recognised to cause diseases that affect neurological, musculoskeletal, gastrointestinal, cardiovascular and renal systems^2–4^ suggesting that it can be a highly invasive pathogen.

*Mycoplasma pneumoniae* is a bacterium of the Mollicutes class, which possesses a small genome of ~ 816 kb that encodes 688 predicted ORFs^5,6^. Similar to other *Mycoplasma* spp., *M. pneumoniae* lacks genes for several metabolic pathways, and for the synthesis of nucleotides, amino acids, lipids and peptidoglycan^6^. *M. pneumoniae* has historically received attention as a model for systems biology research, unique mechanisms for cellular motility, and detailed molecular structural analysis of bacterial attachment organelles^7–12^. *M. pneumoniae* was also one of the first organisms to have its genome annotation interrogated and improved using mass spectrometry data derived from a peptide-centric analysis of the expressed proteome, a method known broadly as proteogenomics^13^.

In *M. pneumoniae*, the major epithelial adhesin P1 protein (MPN141) plays an important role during the early stages of colonisation of the host respiratory tract. It is also essential for motility, a function that is likely to be beneficial during pathogenesis. P1 and its operon partners P90 and P40 (derivatives of MPN142)^14^ are known targets of processing events^15,16^. Recently, we reported comprehensive cleavage patterns and processing sites in P90, P40^17^ and P1^18^. P1 is localised at the terminal region of the attachment organelle and colocalise with P40 and P90^16,19^. Disruption of the *mpn142* gene and the loss of P90 and P40 results in uniform dispersion of P1 over the surface of *M. pneumoniae* and abolition of adherence and virulence^14^. Collectively these observations suggest that processing underpins many essential functions executed by key surface proteins in *M. pneumoniae*.

We recently analyzed the N-terminome and surfaceome of the porcine respiratory pathogen *Mycoplasma hyopneumoniae*^20^, an organism significant for its role as a cause of major economic loss and as a driver of antimicrobial use in pig production^21^. Our studies described a broad range of molecules to be present on the cell surface of *M. hyopneumoniae*, including many proteins with canonical functions in the cytosol that lack obvious signal sequences suggesting they have alternate moonlighting functions^22^. Notably, about half of the detectable proteins found on the cell surface of *M. hyopneumoniae* were targets of proteolytic processing event(s), producing numerous, functionally novel proteoforms^20^. Many processed surface proteins have putative functional interactions with key host extracellular matrix (ECM) molecules, indicating that protein function, pathogenesis and ultimately the development of efficacious vaccines are influenced by proteolytic processing events that remain ill defined.

It is notable that while *M. pneumoniae* has been the subject of intensive study as a model minimal genome bacterium, there have been no systematic analyses of proteolytic protein processing, nor studies that have sought to define the comprehensive repertoire of surface proteins of this important human pathogen. In this study we characterised the surfaceome of *M. pneumoniae* which is the primary interface between pathogen and host, offering key insights about proteins that may interact with the human host, participate in biofilm formation, and bind to inert surfaces. We also conducted a systems wide analysis of protein processing (N-terminomics) in *M. pneumoniae* and cross referenced the two datasets to determine which surface proteins undergo processing. Similar to *M. hyopneumoniae*, we found *M. pneumoniae* uses proteolysis to produce multiple proteoforms from its minimal genome thus expanding the functional proteome. Enrichment strategies using affinity chromatography columns containing actin, fetuin, fibronectin, heparin, plasminogen and cell surface complexes of human lung carcinoma cells (A549) as bait were used to investigate the potential functional repertoire of *M. pneumoniae* cell surface proteoforms. Here we provide an in-depth analysis of two cleaved *M. pneumoniae* surface proteins (MPN052; MPN674); in one instance (MPN674) cleavage fragments retained binding affinities of the parent molecule, while in the other, the fragments gained binding affinities absent in the parent molecule. These observations have significant ramifications for defining the functional proteome of *M. pneumoniae,* understanding pathogenesis and informing subunit vaccine design.

## Results

### N-terminal sequences of *M. pneumoniae* proteins

**Figure 1:**
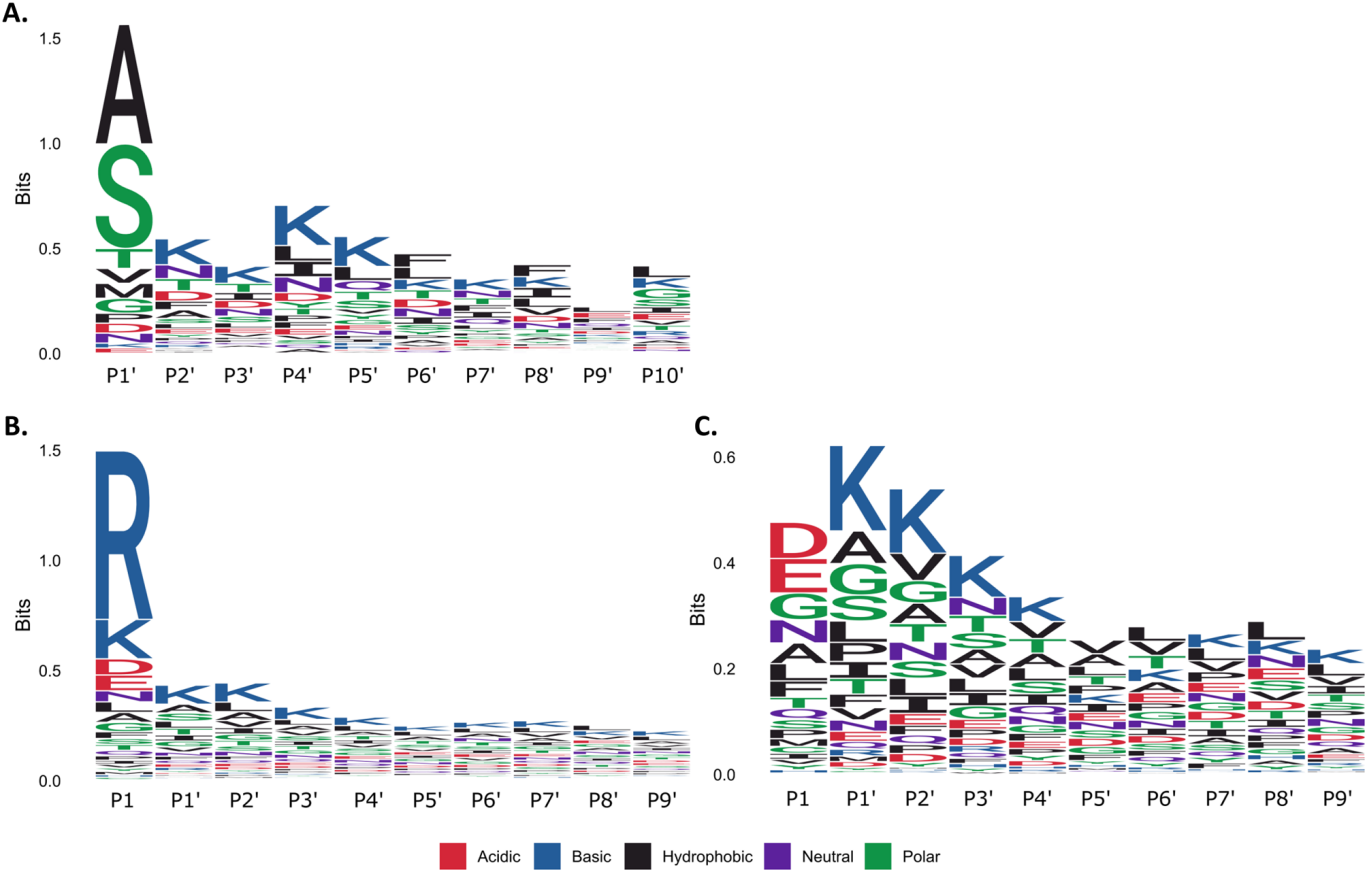
Proteolytic sequence logos of the N-terminome of *Mycoplasma pneumoniae* at (**A**) the protein N-terminus after initiating methionine removal (N-end rule), (**B**) internal neo-N-term endoproteolytic sites and (**C**) N-term endoproteolytic sites excluding tryptic-like sites.

In this study a global analysis of N-terminal peptides was performed using reductive dimethyl labelling of intact *M. pneumoniae* proteins followed by the generation of tryptic peptides and analysis by shotgun LC–MS/MS. This technique allows an untargeted analysis of mature protein sequences, using a chemical tag to identify the first amino acid of the sequence. The sequence is then mapped back to the original ORF, identifying proteins which have been modified post-translationally. Using this method, we obtained 4898 unique N-terminal peptide sequences that mapped to 391 predicted *M. pneumoniae* proteins (Supplementary Data 1). Analysis of the N-termini mapping to positions 1 and 2 of their respective ORFs demonstrated that classical N-end rules^23^ apply in *M. pneumoniae* (Fig. 1A; Supplementary Data 1); with the identification of 163 M*. pneumoniae* proteins beginning with the predicted initiating methionine (iMet) residue. Consistent with the N-end rule, bulky or charged residues in positions 2 and 3 were present in these *bona fide* N-termini, preventing the removal of the iMet by methionine aminopeptidase (MAP). We also identified the removal of the iMet in 66 proteins where the small amino acids alanine, serine and threonine predominated in positions 2 and 3 (Fig. 1A; Supplementary Data 1), with proteins undergoing formyl group removal followed by methionine (Met) excision by MAP, generally when the amino acid in P1′ position is small and uncharged^24^.

Dimethyl labelled neo-N-termini derived from *M. pneumoniae* were mapped beyond position 2 for 317 proteins, indicating that nearly half (46%) of the predicted ORFs (688) of *M. pneumoniae* (strain M129) are post-translationally modified by proteolytic processing (Supplementary Data 1). In addition, 1199 semi-tryptic (unlabelled) peptides were identified, a subset of which may also indicate proteolytic processing has occurred in an additional 27 proteins, totaling 344 ORFs or 50% of the predicted proteome (Supplementary Data 1). An analysis of the N-terminome found that internal neo-N-term endoproteolytic cleavage predominately occurred at tryptic-like sites (Fig. 1B), though many endoproteolytic processing events also occurred when negatively charged residues were in P1′ (D and E) and lysine or serine/alanine in P2′ and P3′ positions (Fig. 1C). Notably two *M. pneumoniae* surface proteins, MPN083 and MPN592, that contain putative peptidase domains (DUF31) related to trypsin peptidases^25^ (Supplementary Data 2) were identified here. These two proteins may be, at least partially, responsible for the most common endoproteolytic event observed—when arginine (R) was in P1 (Fig. 1B).

### The surfaceome of *Mycoplasma pneumoniae*

**Figure 2:**
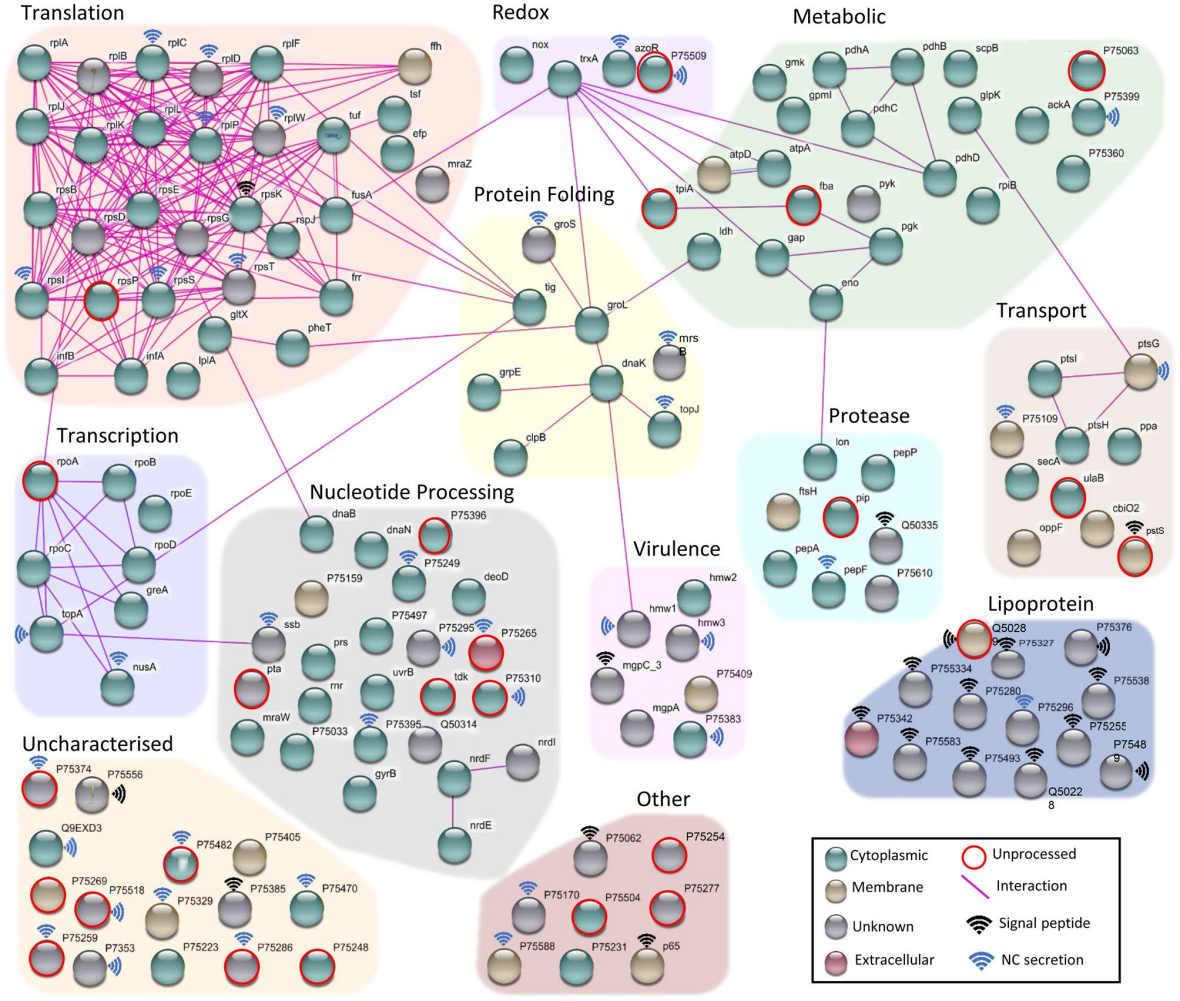
Interaction map of all proteins identified on the surface of *Mycoplasma pneumoniae*, grouped by gene ontology. Proteins with known interactions provided by STRING, are indicated by connecting lines, representing possible protein networks on the cellular surface. Proteins are coloured by their predicted cellular location: green (cytoplasm), brown (membrane), grey (unknown), red (extracellular). Proteins with signal icons either contained a signal peptide (black signal icon) or were found to be non-classically secreted (blue signal icon). Proteins indicated with a red circle were not found to be processed in our N-terminome, all other proteins were found with endo-proteolytic processing sites.

We identified 160 proteins on the surface of *M. pneumoniae* (Fig. 2). Two orthogonal methodologies, trypsin shaving and surface biotinylation that have been used previously by our group to determine surface accessibility showed a 72% overlap (116/160 proteins), in part cross validating these identifications. Biotin enrichment produced the most identifications with 147 proteins while the trypsin shaving experiments identified 127 proteins (Supplementary Data 3). The proteins identified were from a diverse array of classes including lipoproteins, transporters, and adhesins such as P1 (Fig. 2). Interestingly, more than half of the surface proteins (~ 58%) were annotated as having canonical functions in the cell cytosol and 56 (57%) of these have known or predicted interactions with a wide variety of other surface-associated proteins of both similar and disparate functional groups (Fig. 2). Bioinformatic predictions by SignalP^26^ identified signal peptides in 21 proteins (13%), while SecretomeP^27^ accounted for the export of 63 proteins (39%). TMPred^28^ was able to predict one or more transmembrane domains in 74 proteins (46%) (Supplementary Data 3).

### Endoproteolytic processing and putative functions of *M. pneumoniae* surface proteins

**Figure 3:**
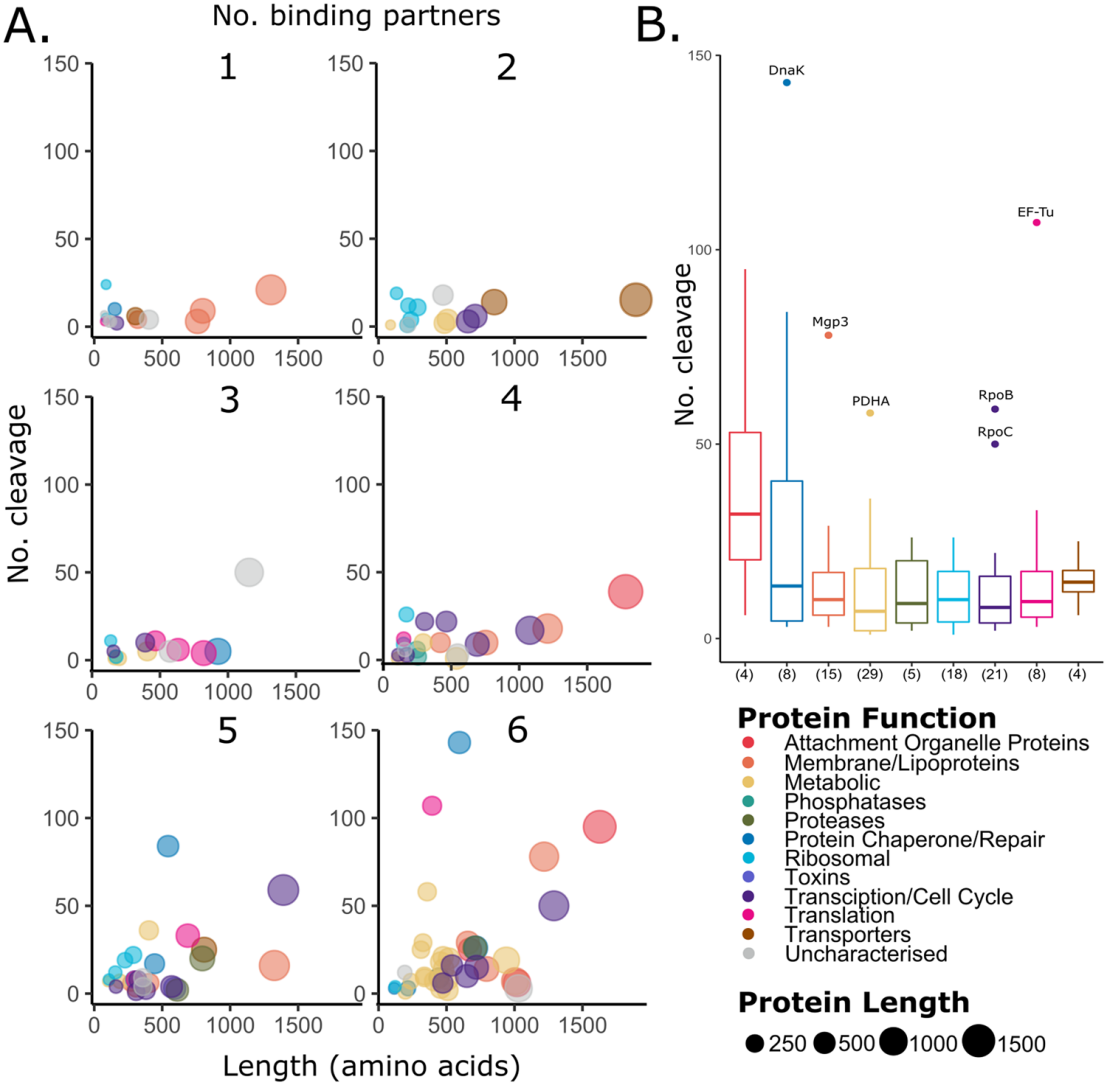
Endoproteolytic processing and putative binding interactions of *M. pneumoniae* surface proteins. (**A**) Multi-panel bubble chart displaying the relationship between protein length and number of cleavage, split by the number of binding partners (1 to 6 ECM molecules). (**B**) Boxplot showing number of cleavages observed in each functional group. The number of proteins assigned to each group is shown in brackets.

When we compared the identities of *M. pneumoniae* proteins found in our surfaceome with the proteins identified by N-terminomics, we identified 134 surface proteins that were also a target of endo-proteolytic processing (Supplementary Data 2). The average number of cleavage events occurring in *M. pneumoniae* surface proteins was 15 per protein, however the number of endoproteolytic sites varied within different protein functional groups, with attachment organelle proteins exhibiting more cleavage events compared to other groups (Fig. 3B). To assign putative functional data to the surface proteins undergoing endoproteolytic events, affinity chromatography, using A549 cells, actin, fetuin, fibronectin, heparin and plasminogen as bait, was used to enrich *M. pneumoniae* protein lysates in an untargeted, systems-wide manner. Of the 134 *M. pneumoniae* surface proteins that are targets of cleavage, 130 were ascribed putative binding interactions. *M. pneumoniae* surface proteins were identified in columns coupled with heparin the most (86%), followed by actin (80%), fibronectin (72%), A549 cells (62%), fetuin (56%) and lastly plasminogen (52%) (Supplementary data 4). We also observed a slight trend in which a greater number of cleavage events was associated with an increase in the number of ECM binding partners (Fig. 3A). This trend was substantiated by a Spearman rank correlation test (rho = 0.3; p-value = 0.00024; scatterplot in Supplementary data 4). The size of the surface protein was not significantly associated with more cleavage events or number of binding partners (Fig. 3A).

### Bioinformatic and proteoform analysis of lipoprotein MPN052

To obtain a more in-depth analysis of proteolysis of surface proteins with known translocation mechanisms, we selected the putative lipoprotein MPN052 (Uniprot ID: P75062). The MPN052 ORF encodes a 657 amino acid protein of 71.7 kDa with an isoelectric point (p*I*) of 9.3. According to the Prorule annotation rule PRU00303, the cysteine residue located at position 27 is the predicted site for attachment of glyceride fatty acid lipid that anchors the lipoprotein to the lipid membrane, presumably on the extracellular surface (see below). Thus, the first 26 amino acids are removed (^1^MFGKGLVKKSLLFFSGVSTMAVFLVSC^27^) in MPN052 indicating the mature protein N-terminus begins at the lipid-modified cysteine residue at position 27. The signal peptide sequence was not identified in any of our datasets. TMPred identified three possible transmembrane domains, 12–28 (Score: 1797), 183–203 (Score: 583), 220–244 (Score: 780). The VSLb2 algorithm identified found two major regions of protein disorder residing between amino acids 253–319 (Score: 0.8241) and 454–621 (Score: 0.8598).

The putative lipoprotein MPN052 was previously found at a molecular weight of ~ 20 kDa and p*I* of ~ 8 by 2D SDS-PAGE^15^, suggesting that cleaved proteoforms are generated for this ORF. We were able to identify the full-length MPN052 on 1D SDS–polyacrylamide gel from a slice representing 50–75 kDa. MPN052 fragments were also identified on the bacterial cell surface by our biotinylation method but was not found in our trypsin cell surface shaving experiments. Our N-terminome data identified 16 unique dimethyl labelled N-termini providing evidence for the exact locations of 4 major endoproteolytic processing sites, as well as a number of minor cleavage events and neo-N-terminal amino peptidase events that sequentially remove single amino acids after the dominant proteolytic cleavage events (Supplementary Data 5). The clipping of amino acids around dominant cleavage events has previously been observed in *M. pneumoniae*^17,18,29^ and *M. hyopneumoniae* where several surface accessible aminopeptidases have been extensively characterised^30–32^. Our surfaceome studies identified several surface accessible peptidases including, MPN197 (oligoendopeptidase F homolog), MPN022 (putative proline iminopeptidase), MPN572 (probable cytosol aminopeptidase), MPN470 (putative Xaa-Pro aminopeptidase), and the two aforementioned putative proteases with trypsin-like domains, MPN592 and MPN083.

**Figure 4:**
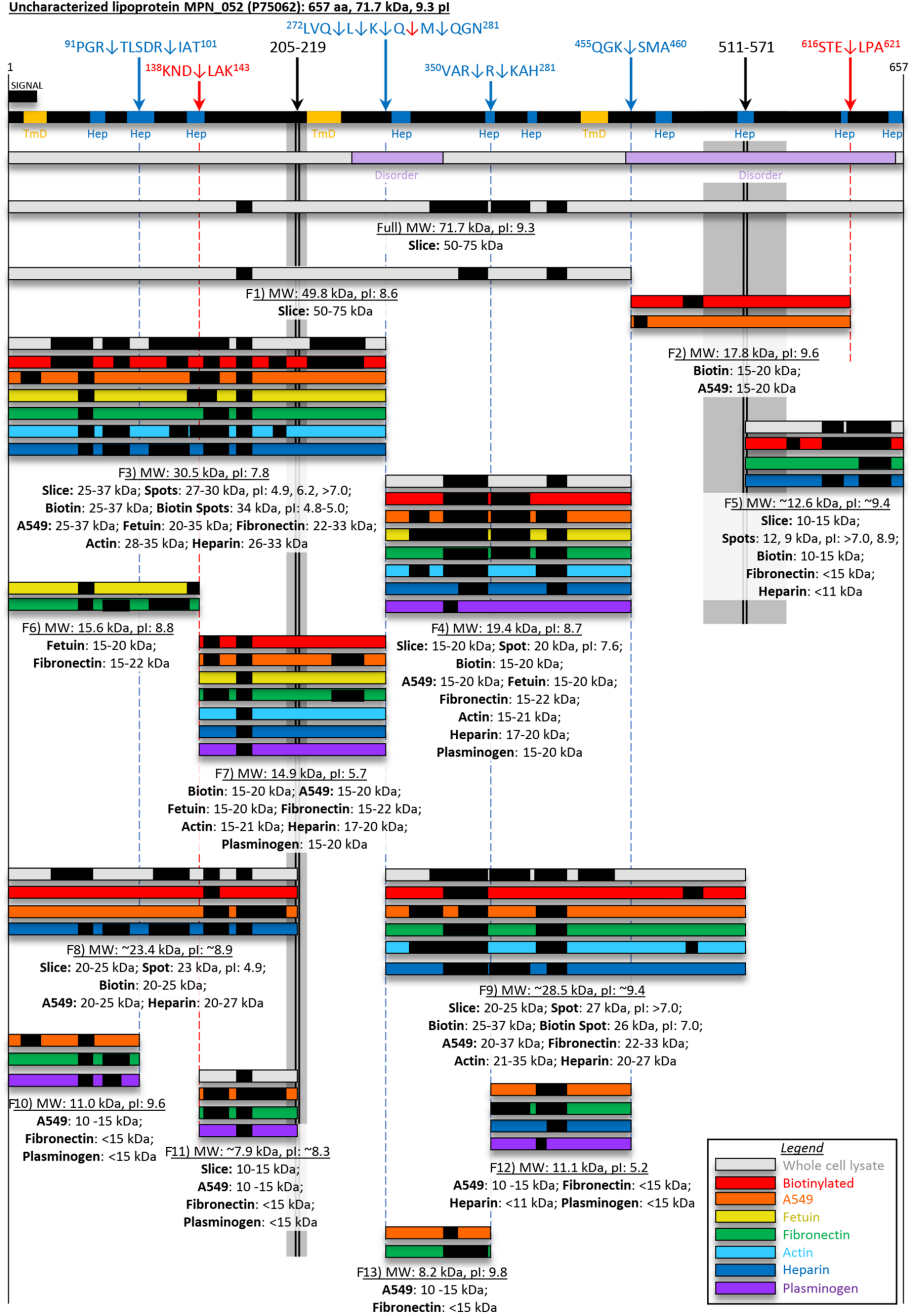
Cleavage map of Uncharacterized lipoprotein MPN052 (P75062). Full length protein designated as “Full” and cleavage fragments are designated F1–F13 as indicated under each set of bars. Cleavage sites identified from N-term dimethyl labelling are indicated by the blue arrows (and blue broken lines) with exact sites shown in the sequence above. Semi-tryptic peptides were also characterised for cleavage sites indicated by the red arrows (and red broken lines). Two hypothetical cleavage sites (black arrows) that span regions 205–219 and 511–571 are shown as the grey boxes, the exact cleavage site is unknown and thus a range is shown. A signal peptide predicted by SignalP is shown. Bioinformatic tools to predict transmembrane domains (TmD, golden boxes), heparin binding domains (Hep, blue boxes), and disordered regions (Disorder, purple boxes) are shown. The peptides shown as black boxes in coloured bars were identified by mass spectrometry from: 1D- and 2D-SDS PAGE of *M. pneumoniae* whole cell lysates (grey bars), and surface biotinylation (red bars). Peptides are also shown from affinity chromatography of: A549 surface complexes (orange bars), fetuin (yellow bars), fibronectin (green bars), actin (light blue bars), heparin (dark blue bars), and plasminogen (purple bars).

Our combined analysis identified 13 cleavage fragments of MPN052 using LC–MS/MS of SDS-PAGE-fractionated *M. pneumoniae* proteins and N-terminomics (Fig. 4). Seven of the 13 proteoforms of MPN052 were identified in biotin labelling experiments indicating that they are surface accessible, and that post-translational proteolysis likely occurs on the cell surface. The use of affinity chromatography separately coupled to different host matrix molecules including heparin, actin, fibronectin, plasminogen and fetuin, and surface proteins of model host epithelial cells (A594 cells) is a method that has been shown to be effective at isolating protein cleavage fragments^17,18,22,29^. Our approach was effective at identifying cleavage fragments of MPN052 and also provided an opportunity to map putative functionally-important adhesive binding domains.

Two major endoproteolytic sites residing at ^272^LVQ↓L↓K↓Q↓M↓QGN^281^ and ^255^QGK↓SMA^460^ generated multiple proteoforms including one fragment of 49.8 kDa (Fig. 4F1); a C-terminal fragment of 17.8 kDa which was truncated by an additional cleavage event at ^616^STE↓LPA^621^ (detected by a semi-tryptic peptide) (Fig. 4F2), a 30.5 kDa fragment (Fig. 4F3) and a 19.4 kDa fragment (Fig. 4F4). Cleavage fragments F3 and F4 were found on the cellular surface of *M. pneumoniae* and were recovered from affinity columns coupled with diverse host molecules, including fetuin, fibronectin, actin and heparin, indicating adhesive binding domains may be located in these regions of MPN052.

Proteoforms that generated peptide coverage to a C-terminal ~ 12.6 kDa region of MPN052 suggested the existence of one or more hypothetical endoproteolytic sites around amino acids 511–571. However, we were unable to find either labelled or semi-tryptic peptide data to confirm the exact location(s) of the endoproteolytic site(s) that generated the C-terminal fragment (Fig. 4F5). This hypothetical endoproteolytic site also explains the presence of a 28 kDa fragment spanning amino acids between the two cleavage sites (^272^LVQ↓L↓K↓Q↓M↓QGN^281^) and the hypothetical proteolytic region between amino acids 511–571 (Fig. 4F9). The existence of fragment F9 is supported by peptide coverage of a gel slice from an SDS-PAGE gel (20–25 kDa) and as a protein spot from a 2D gel (~ 27 kDa, p*I* > 7.0) (Fig. 4F9).

Affinity chromatography studies proved to be valuable in confirming the existence of further cleavage fragments of MPN052 generated by a cleavage event at ^138^KND↓LAK^143^ located by the identification of an unlabelled semi-tryptic peptide ^141^LAKVGFIFVDGQIEK^155^ and the major cleavage site ^272^LVQ↓L↓K↓Q↓M↓QGN^281^. These cleavage events further processed F3 into two fragments of 15.6 kDa (Fig. 4F6) and 14.9 kDa (Fig. 4F7). F7 was found to be surface accessible by the biotinylation method and was identified by LC–MS/MS analysis of *M. pneumoniae* proteins retained on affinity chromatography columns separately coupled with fibronectin, fetuin, heparin, plasminogen and actin. These observations suggest that F7 displayed accessible linear binding motifs that were available for interactions with each of these molecules. The ability of F7 but not F3 to bind plasminogen suggests that cleavage exposes new protein interaction sites. The identification of F6 in affinity columns loaded with fetuin and fibronectin suggests that at least two binding domains exist in F3 for these host proteins.

The identification of endoproteolytic sites at ^91^PGR↓TLSDR↓IAT^101^, identified in our dimethyl labelled N-terminome, and a hypothetical endoproteolytic site between amino acids 205–219, was consistent with recovery of three fragments F8, F10 and F11 (Fig. 4). F8 spans the N-terminal ~ 24 kDa of MPN052, is surface accessible (biotinylated) and carries accessible binding domains for A594 surface proteins and heparin. Putative binding sites for fetuin and fibronectin appear to be inaccessible in F8. Fragment F10, an N-terminal 11 kDa region, was identified by LC–MS/MS analysis of *M. pneumoniae* proteins retained by affinity columns separately coupled with fibronectin and plasminogen. These data suggest that a putative fibronectin binding domain and putative A594 cell and plasminogen-binding domains that are inaccessible in F6, are exposed in the shorter F10 fragment. Putative fibronectin and plasminogen binding domains are exposed in the ~ 7.9 kDa F11 fragment but appear to be inaccessible in the 23.4 kDa F8 fragment. Interestingly, while A549 surface protein complexes interacted with all three fragments, only F8 bound to the heparin column which contained three intact bioinformatically predicted heparin binding regions, whereas F10 contained only one heparin domain and F11 contained none. In contrast, F8 did not interact with either fibronectin or plasminogen in the columns while the two smaller fragments gained this capability. Two smaller proteoforms, F12 and F13, resulting from the major cleavage site ^272^LVQ↓L↓K↓Q↓M↓QGN^281^ and endoproteolytic sites ^350^VAR↓R↓KAH^356^ and ^455^QGK↓SMA^460^ identified by dimethyl labelled neo-N-termini (Fig. 4), were found to bind to A549 surface protein complexes and fibronectin (F12 and 13) as well as heparin and plasminogen (F12 only). Fragments F3, F4 and F7 displayed the widest range of interactions in our affinity chromatography studies, strengthening the evidence for their existence and function. F9 is a 28.4 kDa central fragment of MPN052 that shares significant sequence overlap with the multifunctional fragment F4. While both fragments are accessible on the surface of *M. pneumoniae* and retain an ability to adhere to A594 surface proteins, fibronectin, actin, and heparin, F9 does not share the ability of F3 to interact with fetuin and plasminogen.

**Figure 5:**
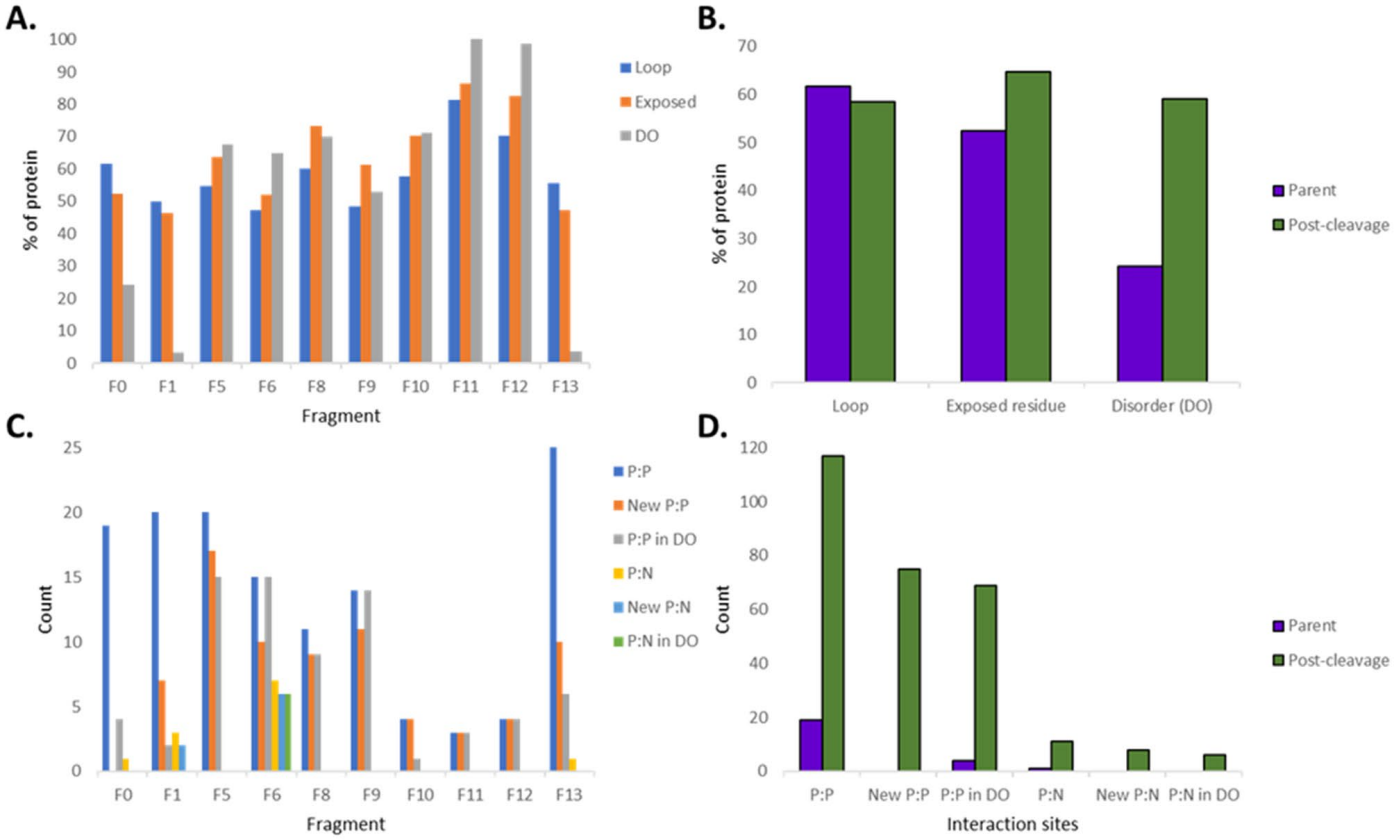
Predicted structural changes to uncharacterized lipoprotein MPN052 (P75062) post-cleavage. (**A**) The percentage of each fragment (F0 = full length) taken up by loop structures, exposed residues, disordered regions (DO). (**B**) Relative change in loop, exposed residues and DO pre- and post-cleavage. (**C**) The number of protein:protein (P:P) and protein:nucleotide (P:N) interaction sites per fragment. (**D**) Relative change in P:P and P:N sites pre and post-cleavage.

In summary, there appear to be five fibronectin, three heparin, three fetuin, three plasminogen, and two actin binding domains in MPN052 which are distributed among the identified proteoforms of MPN052. Some of these proteoforms share partial sequence identity but not putative functionality in our affinity chromatography studies, suggesting structural conformation(s) plays a role in proteoform function. To explore this concept, PredictProtein^33^ was used to predict structural changes between the parent molecule of uncharacterised lipoprotein MPN052 and its fragments. The assessed structural elements included loops, number of surface exposed residues, disordered regions, protein:protein (P:P) interaction sites and protein:nucleotide (P:N) interaction sites. We observed that cleavage fragments were more disordered than the parent (Fig. 5A,B), that new P:P and P:N were predicted in fragments that were absent in the parent molecule (Fig. 5C), and that the majority of these new interaction sites occurred in newly formed disordered regions (Fig. 5D).

### Multiple proteoform analysis of l-lactate dehydrogenase

Glycolytic enzymes have frequently been reported to ‘moonlight’ on the surface of multiple pathogenic and commensal bacteria^34^, and are well described in *M. pneumoniae*^35,36^. Our *M. pneumoniae* surfaceome data identified several enzymes in the glycolytic pathway presented on the cellular surface (Fig. 2). We selected l-Lactate dehydrogenase (LDH_Mpn_) (MPN674/P78007/LDH_MYCPN) for further investigation, as LDH in *M. hyopneumoniae* (LDH_Mhp_) has previously been identified on the bacterial cell surface as a target of multiple processing events^22^. MPN674 encodes a 312 amino acid protein of 33.9 kDa, containing one putative transmembrane domain at its N-terminus, no predicted signal peptide and five predicted putative heparin binding regions. Cell surface trypsin shaving identified LDH peptides spanning the full length of the protein, and regions spanning different parts of MPN674 were identified by LC–MS/MS analysis of biotinylated surface proteins separated by SDS-PAGE.

**Figure 6:**
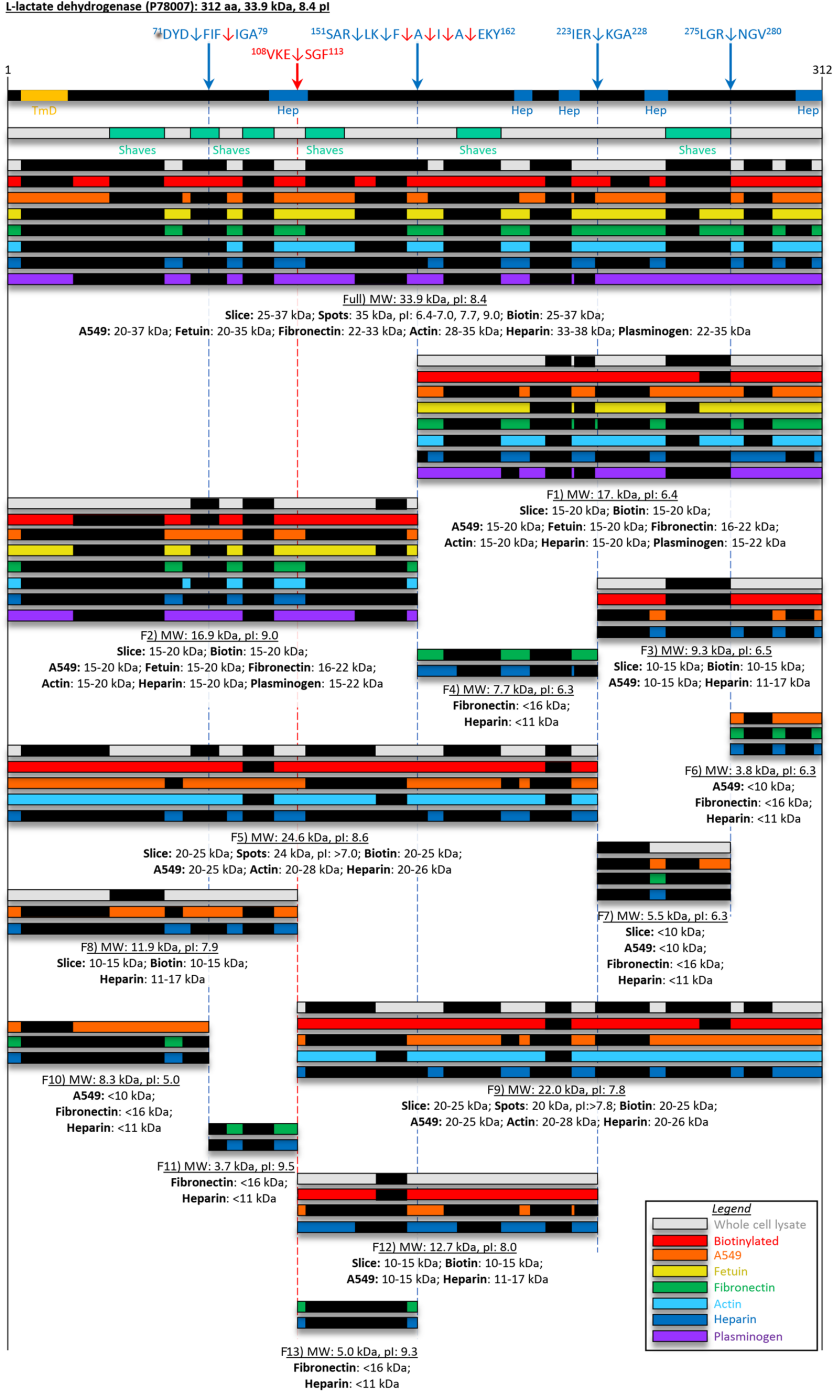
Cleavage map of L-lactate dehydrogenase. Full length protein designated as “Full” and cleavage fragments designated F1–F13, as indicated under each set of bars. Cleavage sites identified from N-term dimethyl labelling are indicated by the blue arrows (and blue broken lines) with exact sites shown in the sequence above. Semi-tryptic peptides were also characterised for cleavage sites indicated by the red arrows (and red broken lines). Bioinformatic tools to predict a transmembrane domain (TmD, golden boxes), heparin binding domains (Hep, blue boxes). The peptides shown as black boxes in coloured bars were identified by mass spectrometry from: 1D- and 2D-SDS PAGE of *M. pnuemoniae* whole cell lysates (grey bars), and surface biotinylation (red bars). Peptides are also shown from affinity chromatography of: A549 surface complexes (orange bars), fetuin (yellow bars), fibronectin (green bars), actin (light blue bars), heparin (dark blue bars), and plasminogen (purple bars).

Eighteen dimethyl labelled neo-N-termini were identified which clustered at four major proteolytic sites (amino acid positions: 74, 154–160, 226 and 278, Fig. 6). The first two cleavage locations were also supported by the identification of semi-tryptic peptides and an additional semi-tryptic cleavage site was identified at ^108^VKE↓SGF^113^.

Unlike MPN052, the full length proteoform of MPN674 was identified in a wide range of our affinity chromatography studies, including fetuin, fibronectin, actin, heparin, plasminogen and A549 surface protein complexes (Fig. 6). Full length MPN674 proteoforms were identified by 2D SDS-PAGE as a train of multiple proteoforms of 35 kDa with p*I*s ranging from of 6.4–9. The central proteolytic cleavage event produces two fragments identified as F1 and F2 that were detected as biotinylated cleavage fragments of 17 kDa. Notably, each fragment retains the ability to bind the same panel of ligands observed for the full length proteoform in affinity chromatography experiments (Fig. 6). The cleavage site ^223^IER↓KGA^228^ produced two fragments, F3 and F4, of 9.3 kDa and 7.7 kDa respectively (Fig. 6). F3 was surface accessible (biotinylated) and displayed the ability to bind A549 surface proteins from A594 cells and heparin while F4 was retained in fibronectin and heparin columns. F5 represents a surface accessible N-terminal fragment of MPN674 of 24.3 kDa. F5 bound to A594 surface proteins, heparin and actin. A cleavage site at ^275^LGR↓NGV^280^ generated two fragments; F6 of 3.3 kDa and F7 of 5.5 kDa. Both F6 and F7 bind surface accessible protein complexes of A595, as well as fibronectin and heparin. A cleavage event at ^108^VKE↓SGF^113^ generated two MPN674 fragments F8 and F9: an 11.9 kDa N-terminal and a 22.0 kDa C-terminal fragment respectively. F8 and F9 were retained in columns of A594 surface proteins and heparin; F9 also bound actin. Two adjacent cleavage sites were identified within F8 ^71^DYD↓FIF^76^ and ^74^FIF↓IGA^79^ producing fragments F10 (8.3 kDa) and F11 (3.7 kDa). Both F10 and F11 retained the ability to bind heparin and gained the ability to bind fibronectin, however only F10 gained the ability to bind A594 surface proteins. Fragment F12 (12.7 kDa) resulted from the cleavage event that generated F9 (^108^VKE↓SGF^113^) and additionally ^223^IER↓KGA^228^. F12 was found to be surface accessible and bound heparin as well as A594 surface protein complexes. Fragment F13 (5.0 kDa) was produced from the cleavage site (^108^VKE↓SGF^113^) and one or more of the central cleavage sites ^151^SAR↓LK↓F↓A↓I↓A↓EKY^162^ and was retained in fibronectin and heparin chromatography.

In summary, six heparin, six fibronectin, two actin and two fetuin binding sites appear to be accessible in the full length proteoform of MPN674.

## Discussion

Proteolysis is one of many post-translational modification (PTM) mechanisms that profoundly influence protein function. Over the past 10 years numerous studies have highlighted how various PTMs impact eukaryote biology. In eukaryotes, protease activity plays a critical role in homeostasis and health by maintaining strict control over protein and cellular function^37–40^. In prokaryote biology, however, systems analysis of proteolysis is in its infancy^41^. Nonetheless, there is sufficient evidence to suggest that controlled proteolysis in pathogenic bacteria belonging to the class *Mollicutes* represents an important mechanism for the generation of multiple proteoforms from a single open reading frame (ORF)^42–51^; underpinning bacterial ectodomain shedding^30,52^ and shaping surface protein architecture^53^. Early studies of bacterial N-terminal sequences by a variety of methodologies suggest that proteolytic processing may be widespread in prokaryote biology^41,54^ but more investigations are required to determine specific mechanisms. Here we used a systems wide N-terminomics methodology to identify N-terminal amino acids in proteins and peptides in *M. pneumoniae*. Our approach identified 4898 unique N-terminal peptide sequences that mapped to 391 functionally diverse *M. pneumoniae* proteins.

In bacteria, formylated methionine (fnMet) initiates protein synthesis. N-terminal methionine excision (NME) is a proteolytic pathway that cleaves the N-termini of proteins, a process that plays a key role in determining how proteins traffic after leaving the ribosome. NME requires the combined actions of peptide deformylase (PDF) and methionine aminopeptidase (MAP) as PDF removes the N-formyl group from the iMet residue (fnMet) enabling MAP to remove the iMet^42^. The precise sequence of amino acids that follow the iMet residue play a role in influencing the efficiency of the cleavage reaction. In *M. pneumoniae*, true N-terminal sequences were characterised for 163 proteins and begin with the initiating methionine (iMet) residue from the predicted ORF. Removal of the iMet was demonstrated in 66 proteins where the small amino acids alanine, serine and threonine predominated in positions 2 and 3 but not when bulky or charged residues were observed in those positions. The strict adherence to the classic N-end processing in *M. pneumoniae* is in stark contrast to what was recently reported in the porcine lung pathogen *M. hyopneumoniae* where atypical N-end processing was observed^20^. Other stark differences in the behaviours of these two pathogens has recently come to light with the observation that *M. hyopneumoniae* but not *M. pneumoniae* lacks dihydrofolate reductase (FolA), methylenetetrahydofolate dehydrogenase (FolD), methionyl-tRNA formyltransferase (FMT) and peptide deformylase (PDF), thus removing all components needed to form, attach, and subsequently remove fnMet^55^. The action of amino peptidases found on the surface of mycoplasmas are known to alter N-termini^31,32^ and this is likely to have occurred with *M. pneumoniae.*

We also identified dimethyl labelled neo-N-termini for 317 *M. pneumoniae* proteins, indicating that nearly half (46%) of the predicted ORFs (688) of *M. pneumoniae* are post-translationally modified by proteolytic processing. Many of these neo-N-termini appear to be attributed to trypsin-like activity although other residues are also targeted. Interestingly, we found two surface-associated *M. pneumoniae* putative proteases (MPN083, MPN592) containing trypsin-like domains (DUF31), which may explain the production of these proteoforms.

To investigate if these neo-N-termini are surface-associated proteoforms we characterised, for the first time, the surfaceome of *M. pneumoniae* and identified 160 proteins. Using two orthogonal methodological approaches we identified enzymes, chaperones, ribosomal proteins, lipoproteins, transporters, adhesins and other attachment organelle proteins on the extracellular surface of *M. pneumoniae*, underscoring the functional diversity of proteins that reside there. More than half of the 160 surface proteins have canonical functions inside the cell and a number of these displayed neo-N-termini that reside well within the true predicted N-terminus indicative of endoproteolytic processing. We were able to link diverse putative binding functions to many of the cleaved proteoforms suggesting that controlled proteolysis represents a mechanism to expand the functional proteome of *M. pneumoniae* by regulating the variety of proteoforms present in the surfaceome/adhesome, without increasing the size of the genome. We found *M. pneumoniae* surface proteins had the highest affinity for heparin, followed by actin, fibronectin, fetuin and then plasminogen. Studies have found that *M. pneumoniae* engages sialyated glycoproteins, such as fetuin and fibronectin, for adherence to respiratory epithelium and for gliding motility^56^ and we recently reported that *M. hyopneumoniae* binds epithelial cell surface actin and that anti-actin monoclonal antibodies are proficient at blocking binding of the organism to porcine-derived PK15 cell monolayers^57^. Our data suggests that *M. pneumoniae* may also be a proficient actin binder, which could potentially contribute to motility and adherence in infection and colonisation. Further studies such as creating recombinants of these proteins and performing ligand–protein assays are needed to examine this hypothesis, as performed in other studies^18,29^.

While protein cleavage is expected to destroy enzymatic function and relieve structural constraint that is inherent and necessary in full length proteoforms, cleaved fragments display increased protein disorder, exposing short linear motifs (SLiMS) that previously were inaccessible and providing greater access to potential PTM and protein:protein interaction sites^58–60^. Indeed, we observed that in general, as the number of cleavage events within a protein increased, so did its ability to associate with more ECM proteins.

Cleavage fragments may execute novel function(s) not displayed in the full length proteoform from which they are derived. It is possible that adhesion is but one function that will be assigned to surface accessible cleavage fragments. Much remains to be learned about the roles played by surface accessible cleavage fragments but we anticipate functional roles in adherence, biofilm formation, and immune evasion, while it is conceivable that smaller cleavage fragments may function as antimicrobial peptides and peptide hormones.

The 160 surface associated proteins we identified in *M. pneumoniae* is a number similar to that reported in *M. hyopneumoniae*^20,22^. While *M. pneumoniae* and *M. hyopneumoniae* share a primary colonisation niche in the respiratory tract, these organisms are phylogenetically and morphologically distinct, host-adapted pathogens and their hosts have different immune response capabilities. We hypothesised that cleavage events in structurally conserved metabolic enzymes that execute the same primary function in both pathogens, but that also moonlight on the cell surface, may undergo different processing events. To test this hypothesis, we compared cleavage sites in lactate dehydrogenase (LDH). Our analyses show that these structurally constrained molecules are tolerant of accumulating mutations in non-conserved regions and that these are different in phylogenetically unrelated pathogens. The full length proteoform of LDH in *M. hyopneumoniae* (LDH_Mhp_) is present on the bacterial cell surface and as two distinct, proteolytic processed proteoforms. LDH_Mhp_ fragments were capable of binding to fibronectin and heparin, whereas the intact full length protein was capable of binding to fibronectin, heparin, actin and PK15 surface proteins^22^. Here, we identified LDH from *M. pneumoniae* (LDH_Mpn_) as a target of multiple proteolytic events. Cleavage sites differed with LDH_Mpn_ centrally cleaved between amino acids ^151^SAR↓LK↓F↓A↓I↓A↓EKY^162^, while LDH_Mhp_ was found to be centrally cleaved between amino acids 188–199 based on peptide coverage in SDS-PAGE slices and affinity chromatography studies^22^. LDH_Mpn_ was also cleaved at 4 other sites which we did not observe in *M. hyopneumoniae*. Proteoforms derived from LDH_Mpn_ were identified in eluents of multiple affinity chromatography experiments, although in most cases the range of binding activities of each proteoform was reduced compared to full length protein, similar to the observations of LDH proteoforms in *M. hyopneumoniae*^22^.

Lipoproteins and adhesins are either fully or partially exposed on the cell surface. These cell-membrane bound proteins are under strong selection pressure and evolve in ways that are unique to the species. Processing events will be guided by the types of proteases found on the cell surfaces of these different mycoplasmas. There is already a precedence for the functional importance of processing of lipoproteins where the release of lipopeptides from the N-terminus of lipoprotein MALP-P has important ramifications for the host immune response^61,62^.

Lipoproteins which reside on the cellular surface of *M. pneumoniae* are known to be important during pathogenesis^63^, particularly as inducers of NF-κB and inflammation in the absence of factors such as lipopolysaccharide (LPS)^64^. However, many *M. pneumoniae* lipoproteins remain uncharacterised and are simply hypothetically predicted sequences using bioinformatics. Here we have characterised the proteolytic cleavage of Uncharacterised Lipoprotein MPN052 (P75062), which our data demonstrates is displayed as 14 distinct proteoforms. We identified multiple sized-fractioned proteoforms that bound host ECM components fetuin, fibronectin, actin, heparin and plasminogen in affinity chromatography columns. It is important to note the reduced binding capacity of the full length proteoform, compared to the post-translationally processed proteoforms, which may be the result of a predicted increase in disordered regions and exposed protein:protein interaction sites post-cleavage.

Most studies that report the presence and potential function(s) of bacterial moonlighting proteins by microbial pathogens use bottom-up mass spectrometry techniques, which detect peptides originating from the predicted protein ORF^34^. This experimental strategy is not capable of definitively discriminating between the peptides originating from a full-length bacterial protein or the peptides of a unique post-translationally processed proteoform from the same ORF. We raise the possibility that some of the literature around moonlighting proteins may in fact be identifications of post-translationally modified proteoforms, rather than identifications of the original protein performing multiple functions.

Although beyond the scope of this work, it would be of great interest to accurately quantitate the abundance of bacterial cell surface proteoforms. Such analyses would provide a better understanding of the proportion of full length ORFs targeted by proteolysis and exposed to the host. Biotinylated surface proteins could be used for this purpose on 2D PAGE, although this approach is likely to underestimate the surfaceome compared to the two bottom-up biotinylation and trypsin shaving strategies utilised here. A more costly TMT or iTRAQ surface labelling approach could also be used to quantitate the surface proteins to cytosolic proteoforms, although incomplete labelling of surface proteins and/or accidental labelling of internal proteoforms due to the highly hydrophobic tags would compromise this type of analysis^65^. It is important to note that the exposure time for the surface labelling and trypsinisation in our methodology is kept short to avoid this problem and therefore likely underestimates the surface proteome.

Our studies have shown that proteolysis is a significant contributor to proteoform diversity in the mycoplasmas, greatly expanding the functional potential of two genome-reduced pathogens. Modification of proteoforms presented on the cell surface of these prokaryotes has consequences for the capacity of putative host–pathogen interactions and may lead to improved understanding of the persistence of these organisms in their respective hosts.

## Materials and methods

### *Mycoplasma pneumoniae* culture conditions

The *M. pneumoniae* (strain M129) cells were cultured in modified Hayflick’s medium at 37 °C in tissue culture flasks using established culture conditions^66^. Modified Hayflick’s medium contained 21 g PPLO broth base without crystal violet, 5 g of d-glucose, 4 mL of 0.5% phenol red, 100 mL of liquid yeast extract (150 g/L), 200 mL heat-inactivated horse serum (56 °C, 30 min) supplemented with 1 g ampicillin [Sigma, A5354, St. Louis, MO, USA] per litre. *M. pneumoniae* cells were harvested at mid-log phase by washing adherant cells 3 times with PBS and collected using cell scaping.

### Dimethyl labeling of *M. pneumoniae* proteins

Dimethyl labeling of *M. pneumoniae* proteins was performed as described previously^20^. Briefly, *M. pneumoniae* cells were resuspended in 6 M guanidine hydrochloride with Complete protease inhibitors [Roche] and lysed with an ultrasonic probe 3 times for 30 s, with 100% duty cycle at 50% power. *M. pneumoniae* protein lysates were reduced using 5 mM tributylphosphine [Sigma-Aldritch] and alkylated with 20 mM acrylamide monomers [Sigma-Aldritch] for 90 min at room temperature. Proteins were precipitated 8 volumes of acetone and 1 volume of methanol at − 80 °C for 3 h, pelleted by centrifugation at 20,000×*g* for 20 min and resuspended in 100 mM HEPES solution adjusted to pH 7. Protein labelling was performed on 1 mg of *M. pneumoniae* (strain M129) protein by the addition of 20 mM formaldehyde (ultrapure grade) [Polysciences, USA] and 20 mM sodium cyanoborohydride [Sigma-Aldritch], every 30 min to a final concentration of 60 mM buffered in a final volume of 1 mL and incubated at 37 °C for a minimum 4 h. The reaction was quenched by the addition of 100 mM Tris for 30 min at 37 °C and then precipitated with 8 volumes of acetone and 1 volume of methanol at − 80 °C for 3 h. The precipitated protein was then pelleted by centrifugation at 20,000×*g* for 20 min and washed with 5 volumes of methanol. The protein pellet was resuspended in 50 mM sodium hydroxide, pH 8.0 and digested with Trypsin Gold [Promega, USA] overnight at 37 °C. The acetonitrile was evaporated using a speedvac, desalted by SiliaPrepX HLB Polymeric SPE cartridges [Silicycle, Canada], and analysed by LC–MS/MS. All experiments were performed in biological triplicates. N-terminomics experiments also performed in technical triplicate.

### *Mycoplasma pneumoniae* enzymatic cell surface shaving

Trypsin shaving of *M. pneumoniae* cells was carried out as described previously^29^. Briefly, freshly harvested *M. pneumoniae* cells were washed extensively (> 3 times) in PBS and pelleted by centrifugation (4000×*g* 10 min 4 °C). Cells were resuspended in PBS (pH 7.8) and enzymatic cell shaving using trypsin was performed at 37 °C for 5 min. Intact cells were pelleted by centrifugation and the supernatant containing liberated surface proteins collected on ice to cease trypsin activity. Surface proteins were analysed by 1D gel electrophoresis or further digested to peptides with trypsin prior to analysis by SCX and LC–MS/MS as described previously^29^. All enzymatic cell shaving experiments performed in biological triplicates.

### Cell surface biotinylation of *M. pneumoniae* proteins

Freshly harvested *M. pneumoniae* cells were washed extensively (> 3 times) in PBS and pelleted by centrifugation (4000×*g* 10 min 4 °C). Cells were resuspended in PBS (pH 7.8) and biotinylated with Sulfo-NHS-LC Biotin (Thermo Scientific) for 30 s on ice. The reaction was then quenched with the addition of a final concentration of 50 mM Tris–HCl (pH 7.4) and incubated for 15 min. Cells were washed in three changes of PBS and pelleted by centrifugation. Cells were lysed and precipitated protein was pelleted, air dried and resuspended in buffers appropriate for downstream application as previously described^53^. Approximately 1 mg of protein was subjected to avidin affinity chromatography in a column packed with 1 ml of immobilised monomeric avidin [Thermo Fisher, USA], separated by 1D-SDS-PAGE into 16 size fractions and analysed on a QSTAR Elite on 60 min gradients. All biotinylation experiments were performed in biological triplicates.

### Preparation of *M. pneumoniae* proteins for 2D gel electrophoresis

*Mycoplasma pneumoniae* cells were extensively washed with PBS and lysed in 7 M urea, 2 M thiourea, 40 mM Tris–HCl, 1% (w/v) C_7_BzO detergent [Sigma], followed by three 30 s rounds of sonication at 60% power on ice. Proteins were reduced and alkylated with 5 mM tributylphosphine and 20 mM acrylamide monomers for 90 min at room temperature. Insoluble material was removed by centrifugation and five volumes of acetone added to precipitate protein. After centrifugation, the protein pellet was solubilised in 7 M urea, 2 M thiourea, 1% (w/v) C_7_BzO for two-dimensional gel electrophoresis^17^.

### Liquid chromatography tandem mass spectrometry (LC–MS/MS)

Using an Eksigent 415 autosampler connected to a 415 nanoLC system (Eksigent, USA), 5 µL (1 μg/μL) of the sample was loaded at 300 nl/min with MS buffer A (2% acetonitrile, 0.2% formic Acid) by direct injection onto a PicoFrit column (75 µmID × 150 mm; New Objective, Woburn, MA) packed with C18AQ resin, 1.9 µm 200 Å [Dr Maisch, Germany]. Peptides were eluted from the column and into the source of a 5600 TripleTOF hybrid quadrupole-time-of-flight mass spectrometer [Sciex, USA] using the following program: 2–35% MS buffer B (80% acetonitrile, 0.2% formic acid) over 90 min, 35–95% MS buffer B over 9 min, 95% MS buffer B for 9 min, 80–2% for 2 min. The eluting peptides were ionised at 2300 V. An Intelligent Data Acquisition (IDA) experiment was performed, with a mass range of 350–1500 Da continuously scanned for peptides of charge state 2+ to 5+ with an intensity of more than 400 counts/s. Up to 50 candidate peptide ions per cycle were selected and fragmented and the product ion fragment masses measured over a mass range of 100–2000 Da. The mass of the precursor peptide was then excluded for 15 s.

### Mass spectrometry data analysis

Mass spectrometry data analysis was performed as described previously^20^. The MS/MS data files produced by the 5600 TripleTOF were searched using Mascot Daemon (version 2.4^67^) against the MSPnr100 database (based on the major reference sequence repositories of NCBI, Refseq, UniProtKB, EupathDB and Ensembl. Duplicate entries at the species-level are removed, “multispecies” entries (NCBI) and “fragments” (TrEMBL) are ignored. The search parameters were: Precursor tolerance 10 ppm and product ion tolerances ± 0.2 Da; charge states: 2+, 3+ and 4+ ; propionamide (C), dimethyl (K) specified as fixed modifications; oxidation (M), deamidation (NQ), Dimethyl (N-term) specified as variable modifications; enzyme specificity was semi-ArgC; 1 missed cleavage was allowed. The results of the search were then filtered by including only peptides with a dimethyl labelled N-terminus and excluding peptides with a p-value greater than 0.05.

### Affinity chromatography of different host molecules

*Mycoplasma pneumoniae* affinity chromatography to different host molecules was performed as described previously^17^. Host proteins used here were: bovine actin [Sigma, code: A3653], purified fibronectin [Merck Millipore, code: 341635], plasminogen from human plasma [Merck Millipore, code: 528175], and bovine fetuin [Sigma, code: F3004] sourced from Sigma. Host proteins were biotinylated using the same protocol as detailed for cell surface proteins above and bound to Pierce Avidin Agarose beads [Thermo Scientific]. *M. pneumoniae* cells were lysed in 1% (w/v) C7BzO [Sigma-Aldrich] in PBS (pH 7.8) and incubated with the host protein-Avidin Agarose mixture. Non-bound *M. pneumoniae* protein complexes were washed with PBS and bound complexes were eluted with 7 M urea, 2 M thiourea, 40 mM Tris–HCl, and 1% (w/v) C7BzO. Elutions were separated by 1D-SDS PAGE and proteins were identified by LC–MS/MS. Human lung carcinoma (A549, ATCC CCL-185) cells were cultured in RPMI 1640 medium [Invitrogen] supplemented with 10% heat inactivated fetal bovine serum. Cells were grown in tissue culture flasks at 37 °C with 5% CO_2_. Cells were biotinylated, washed, and lysed in 1% (w/v) C7BzO [Sigma-Aldrich] in PBS (pH 7.8). Like above, these biotinylated A549 proteins were incubated with Pierce Avidin Agarose beads. Mixing, washing, and eluting *M. pneumoniae* proteins complexes was performed as described above. Heparin affinity chromatography was performed with a HiTrap Heparin HP column [GE Healthcare]. *M. pneumoniae* cells were lysed in 10 mM sodium phosphate, 0.1% Triton TX-100 (pH 7.0) and protein complexes were loaded onto the HiTrap column. Unbound complexes were washed with 10 mM sodium phosphate (pH 7.0) and bound complexes were eluted in 10 mM sodium phosphate (pH 7.0) with increasing concentrations of sodium chloride (up to 2 M). Elutions were separated by 1D-SDS PAGE and proteins were identified by LC–MS/MS.

### Bioinformatic and statistical analyses

The putative functions of *M. pneumoniae* were assigned by the Uniprot database and predicted cell localisations were determined by PSORTb^68^ and SecretomeP^27^. Transmembrane domains were predicted by TMPred^28^ and the presence of signal peptides was predicted by SignalP^26^. Protein interaction networks were predicted using STRING^69^. Sequence logos were generated using R packages ggplot2 and ggseqlogo^70^. Spearman’s rank correlation coefficient and scatterplot were generated using R package ggpubr and the cor.test() and ggscatter functions.

## Supplementary information

Supplementary Table S1.

Supplementary Table S2.

Supplementary Table S3.

Supplementary Table S4.

Supplementary Table S5.

Supplementary Figures.

## Data Availability

All mass spectrometry proteomics data generated by this study have been deposited to the ProteomeXchange Consortium via the PRIDE^71^ partner repository with the dataset identifier PXD022356.
