# Supplementary material for: Protein cleavage influences surface protein presentation in Mycoplasma pneumoniae
Source: Sci Rep. 2021 Mar 24;11:6743. doi: 10.1038/s41598-021-86217-y (PMC7990945; doi:10.1038/s41598-021-86217-y)
Supplement: Supplementary file 6 — Supplementary Figures. [file 41598_2021_86217_MOESM6_ESM.pdf]

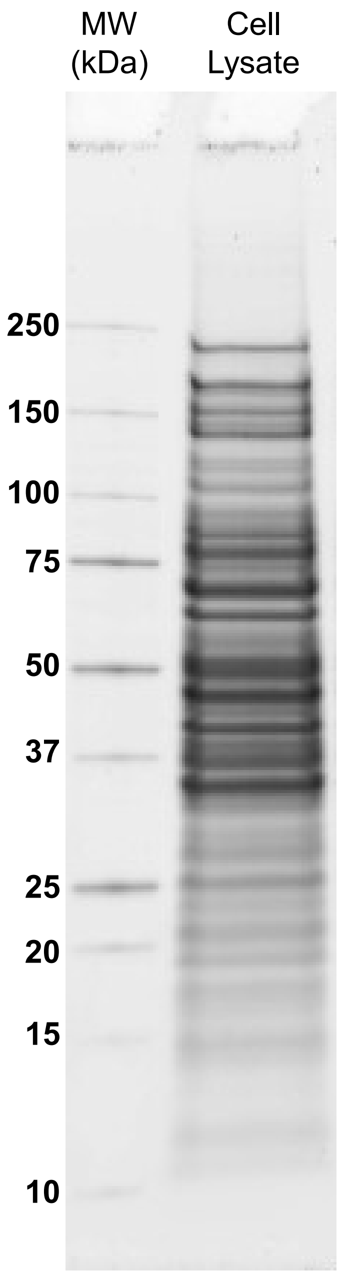

Figure 1: One-Dimensional SDS-PAGE of *M. pneumoniae* whole cell lysate.

M. pneumoniae cells were harvested and lysed in 7 M urea, 2 M thiourea, 1% (w/v) C7BzO and subjected to One-Dimensional SDS gel electrophoresis as described in materials and methods.

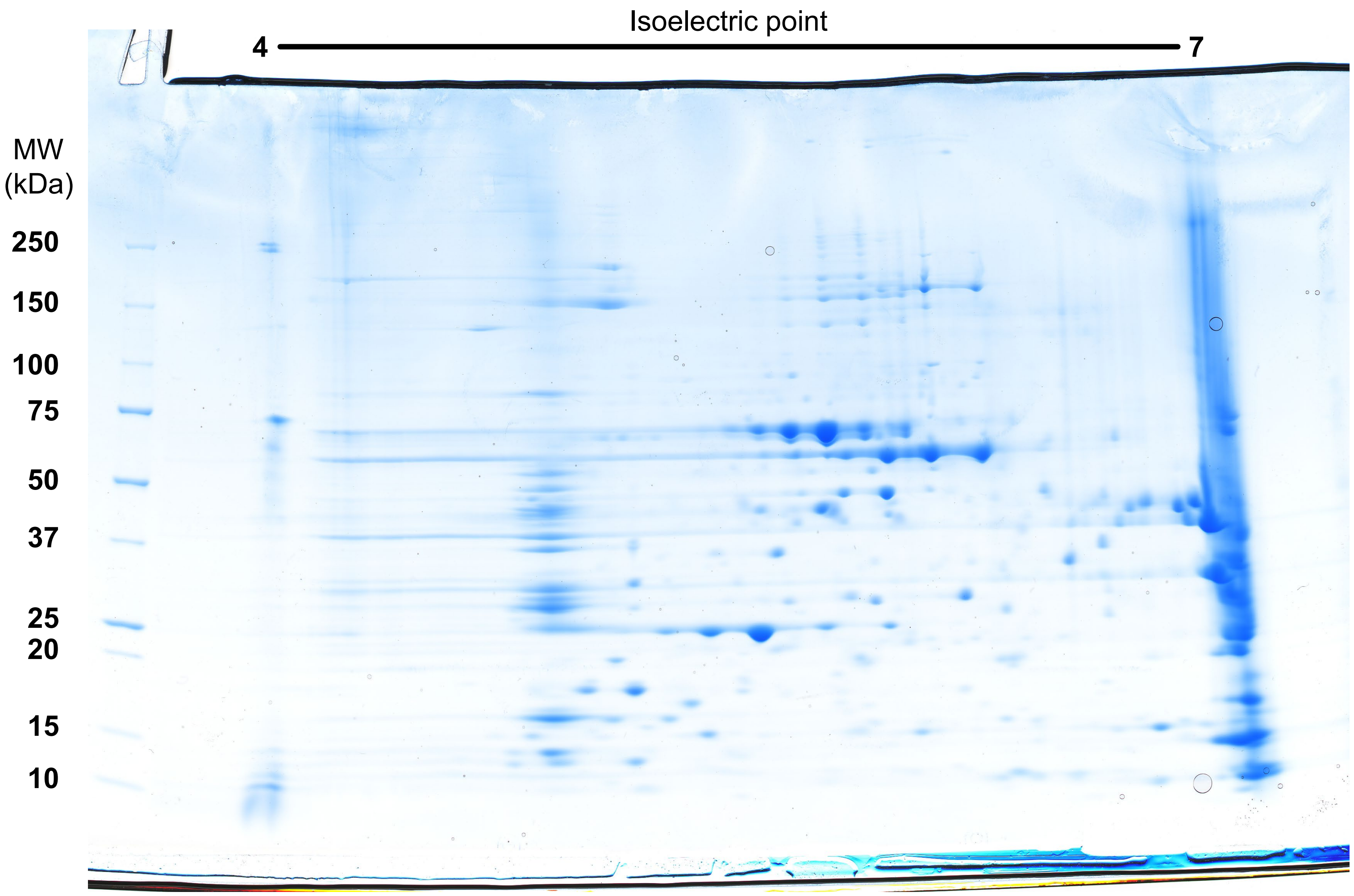

Figure 2: Two-Dimensional SDS-PAGE (4 – 7 pI) of *M. pneumoniae* whole cell lysate.

M. pneumoniae cells were harvested and lysed in 7 M urea, 2 M thiourea, 1% (w/v) C7BzO. Soluble proteins were separated by isoelectric point on a pH 4 – 7 gel strip in the first dimension, followed by mass on SDS PAGE in the second dimension.

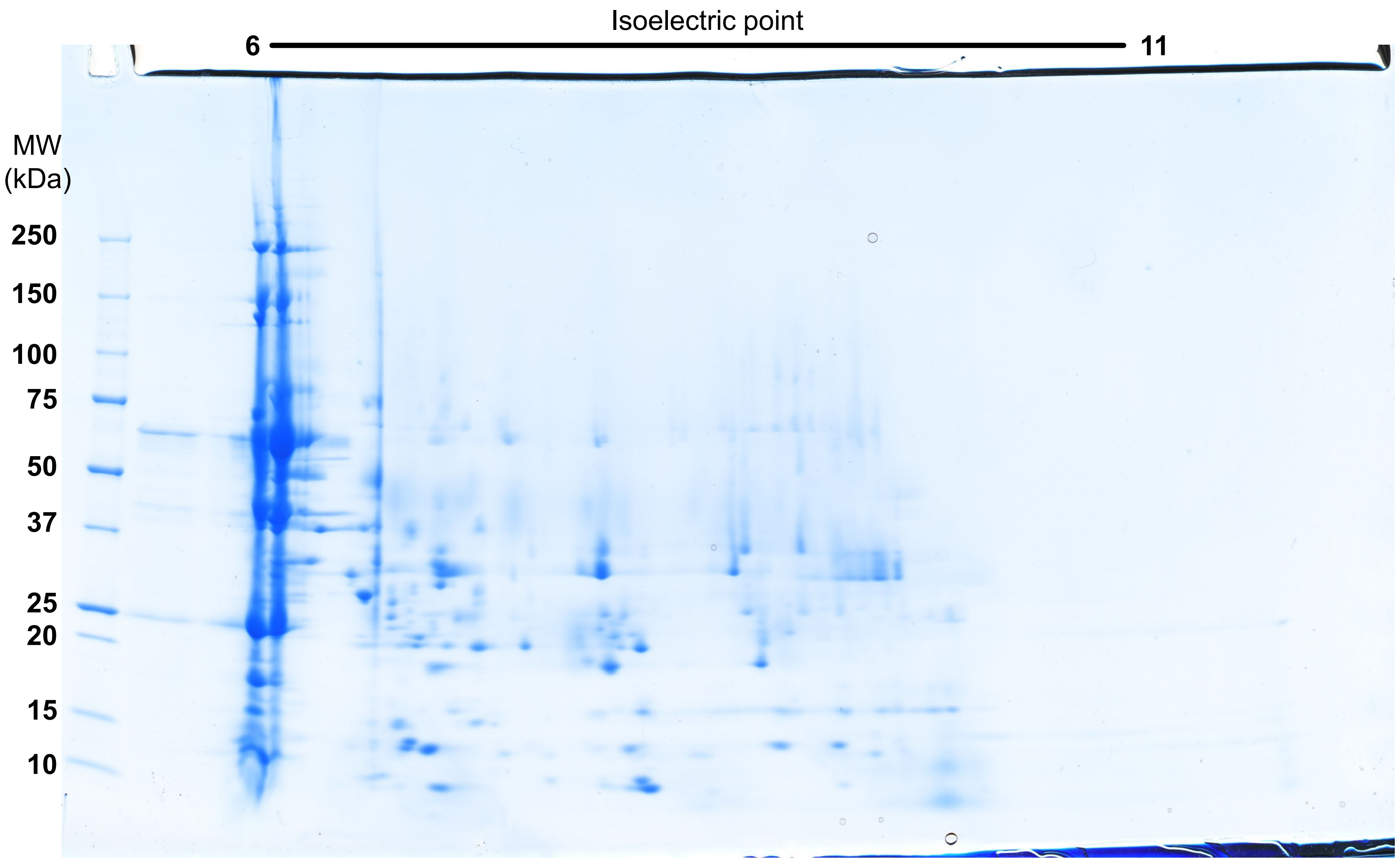

Figure 3: Two-Dimensional SDS-PAGE (6 – 11 pI) of *M. pneumoniae* whole cell lysate.

M. pneumoniae cells were harvested and lysed in 7 M urea, 2 M thiourea, 1% (w/v) C7BzO. Soluble proteins were separated by isoelectric point on a pH 6 – 11 gel strip in the first dimension, followed by mass on SDS PAGE in the second dimension.
